# Designing a Virtual Reality Game for Promoting Empathy Toward Patients With Chronic Pain: Feasibility and Usability Study

**DOI:** 10.2196/17354

**Published:** 2020-08-07

**Authors:** Xin Tong, Diane Gromala, Seyedeh Pegah Kiaei Ziabari, Christopher D Shaw

**Affiliations:** 1 Simon Fraser University Surrey, BC Canada

**Keywords:** virtual reality, serious games, empathy, chronic pain, game design

## Abstract

**Background:**

Many researchers have been evaluating how digital media may impact the emotional and perspective taking aspects of empathy in both clinical and nonclinical settings. Despite the growing interest in using virtual reality (VR) and VR games to motivate empathy, few studies have focused on empathy for people who live with chronic pain.

**Objective:**

Chronic pain affects, by conservative estimates, 1 in 5 people in industrialized countries. Despite this prevalence, public awareness of chronic pain was remarkably low until the recent opioid crisis; as a result, stigma remains a problem frequently faced by people who live with this condition. To address this, the VR game *AS IF* was developed to increase nonpatients’ empathy toward the growing number of people who live with long-term chronic pain. On the basis of our prior work, we overhauled our approach, designed and built a VR prototype and evaluated it, and offered design suggestions for future research.

**Methods:**

We introduced the design features of the VR game *AS IF* and described the study we devised to evaluate its effectiveness. We adopted a mixed methods approach and compared the empathy-related outcomes in both pre- and posttesting. A total of 19 participants were recruited.

**Results:**

The findings of this study suggest that the VR game was effective in improving implicit and explicit empathy as well as its emotional and perspective taking aspects. More specifically, for the *Empathy Scale*, the total pretest scores (mean 47.33, SD 4.24) and posttest scores (mean 59.22, SD 4.33) did not reach statistical significance (*P*=.08). However, we did find differences in the subscales. The *kindness* subscale showed a statistically significant increase in the posttest score (mean 15.61, SD 2.85) compared with the pretest score (mean 17.06, SD 2.65;*P*=.001). For the *Willingness to Help Scale*, a significant increase was observed from a t test analysis (*P*<.001) of scores before (mean 7.17, SD 2.28) and after (mean 8.33, SD 2.03) the gameplay. The effect size for this analysis was large (*d*=−1.063).

**Conclusions:**

The contributions of this research are as follows: *AS IF* provides a promising approach for designing VR games to motivate people’s empathy toward patients with chronic pain, the study evaluates the potential effectiveness of such a VR approach, and the general design suggestions devised from this study could shed light on future VR game systems.

## Introduction

### Background

Pain is a basic and necessary experience that alerts us to physical harm or infection. However, pain is notoriously difficult to describe, and it is harder for one person to understand what another person’s pain is like. The International Association for the Study of Pain defined chronic pain as pain that persists for more than 3 months [1], whereas acute pain refers to pain that persists for a shorter time and is expected to subside with healing (eg, pain from surgery or childbirth) [2]. Although most people have experienced acute pain, chronic pain persists well beyond the expected time of healing and can last a lifetime [3]. Although chronic pain is now considered to be a condition [1], its cause and cure have yet to be discovered, and it has no clear biomarkers. In addition, chronic pain often has associated sequelae, from anxiety, depression, insomnia, and cognitive impairment to decreasing mobility [3].

Conservative estimates suggest that 1 in 5 people of all ages live with chronic pain [1,3]. Despite this high prevalence, people who live with chronic pain also face social stigma and high rates of social isolation [4]. More than loneliness, social isolation is correlated with a decreasing quality of life and earlier morbidity [3,5]. As treating this long-term pain means managing it, it is important that caregivers and health professionals find ways to understand the *lived experience* of chronic pain sufferers—in other words, what it is like to actually live with the debilitating effects of long-term pain and how it impacts a patient’s biopsychosocial realities, their ability to function, and their quality of life [3].

Definitions of empathy are wide ranging: the most common is “our ability to perceive, understand, and respond to the experiences and behavior of others” [6]. According to Davis [7], empathy has 3 primary dimensions: (1) physical sensations, (2) emotional sensations, and (3) cognitive awareness (perspective taking). In many studies from the medical and cognitive science domains, the emotional and perspective taking dimensions of empathy were the predominant dimensions that were evaluated, along with their effects in both clinical and nonclinical environments [8-13].

However, despite renewed attention on empathy, some studies found that teaching empathy has declined in undergraduate education both in medical schools [8,14,15] and colleges in general [16]. Researchers believe that we can mitigate this situation by teaching empathy in medical schools by *embedding it in students’ actual experiences with patients* [17], which can lead to better diagnoses [17-19]. Consequently, empathic communication may help patients manage their health by fostering trust between them and their health professions [9]. Hence, empathy training may similarly improve the attitudes of nonpatients toward patients with chronic pain. (Here, we define *nonpatients* in terms of 2 populations: (1) doctors, nurses, and health professionals who work with patients with chronic pain and (2) caregivers and family members of chronic pain sufferers.)

Previous studies demonstrated that games and video games have a high potential for fostering empathy toward a certain population, even if the players are concerned about winning the game [20-24]. The sense of embodiment in virtual reality (VR) games can appear significantly more effective compared with watching a 2D video [12,25,26]. Evidence also showed that VR games and environments can facilitate a significantly higher level of empathy than videos or traditional media forms [27]. The immersive and convincing nature of VR has profound effects and may confer meaningful benefits for an individual’s cognition or behavior [28]. For instance, Bailenson et al [27] found that subjects showed a higher occurrence of thinking from the place of their partners in a VR perspective taking experience. They also observed an improvement in participants’ empathy levels toward their partners when they considered the patients’ perspective [27]. In other words, two experiential aspects of VR—an immersive sense and an embodied sense—appear to play important roles in empathy [27,29,30].

### Objectives

To develop or strengthen a more empathic connection between nonpatients and patients with chronic pain, we designed an interactive VR game entitled *AS IF* [31]. In this VR game, participants *inhabit* an avatar—a 3D character who has chronic pain—from a first-person perspective. Participants were then asked to perform a series of activities and tasks as patients with chronic pain. In other words, *AS IF* was developed to enable participants to not only inhabit the virtual body of a patient with chronic pain but also to metaphorically *walk in the shoes* of this patient by experiencing physical limitations during tasks and to hear that patient’s self-talk during each task. We designed *AS IF* with a view to eventually deploying it for clinicians and caregivers. Overall, the goals of this study were to (1) increase empathy toward the growing number of people who live with chronic pain, (2) evaluate the prototype, and (3) offer design suggestions that may guide future research.

## Methods

### *AS IF*, the Game Design

Initially, a non-VR desktop version of *AS IF* was developed and tested by the authors using *Microsoft Kinect* [31]. Later, we conducted a study to evaluate the effectiveness of this interactive game system [32]. In that version [31,32], the game tasks involved completing *connect-the-dots* puzzles from a third-person perspective. The player *inhabited* and could move the limbs and head of a virtual avatar—a grandmother living with chronic pain. Players listened to the grandmother’s self-talk while solving puzzles; each solved puzzle resulted in the completion of a kitchen task. From analyzing the feedback collected in playtesting, we found that participants were more willing to help people with chronic pain and exhibited positive attitudes about playing from the first-person perspective. However, participants wanted to interact with virtual objects more directly, rather than solving the more abstract connect-the-dots puzzles, and some suggested we design a comparable game in VR for that reason.

Therefore, we redesigned the game and switched from *Microsoft Kinect’s* platform to an immersive VR platform, the *HTC VIVE* [33]. In addition, we made significant changes to the game using the *Unity3D* [34] game engine. Here, we outline the revision of *AS IF*.

In this VR version, from the first-person perspective, the player inhabits an avatar of a grandmother who lives with chronic pain. Participants attended to tasks that required physical movements and, as the grandmother, experienced limitations of those movements as she would, a point that was made in her *self-talk* or audible narration. The embodiment aspects of the VR game feature multimodal feedback; design decisions such as this were based on the theory of embodied simulation [35].

#### Narrative and Stories

**Figure 1:**
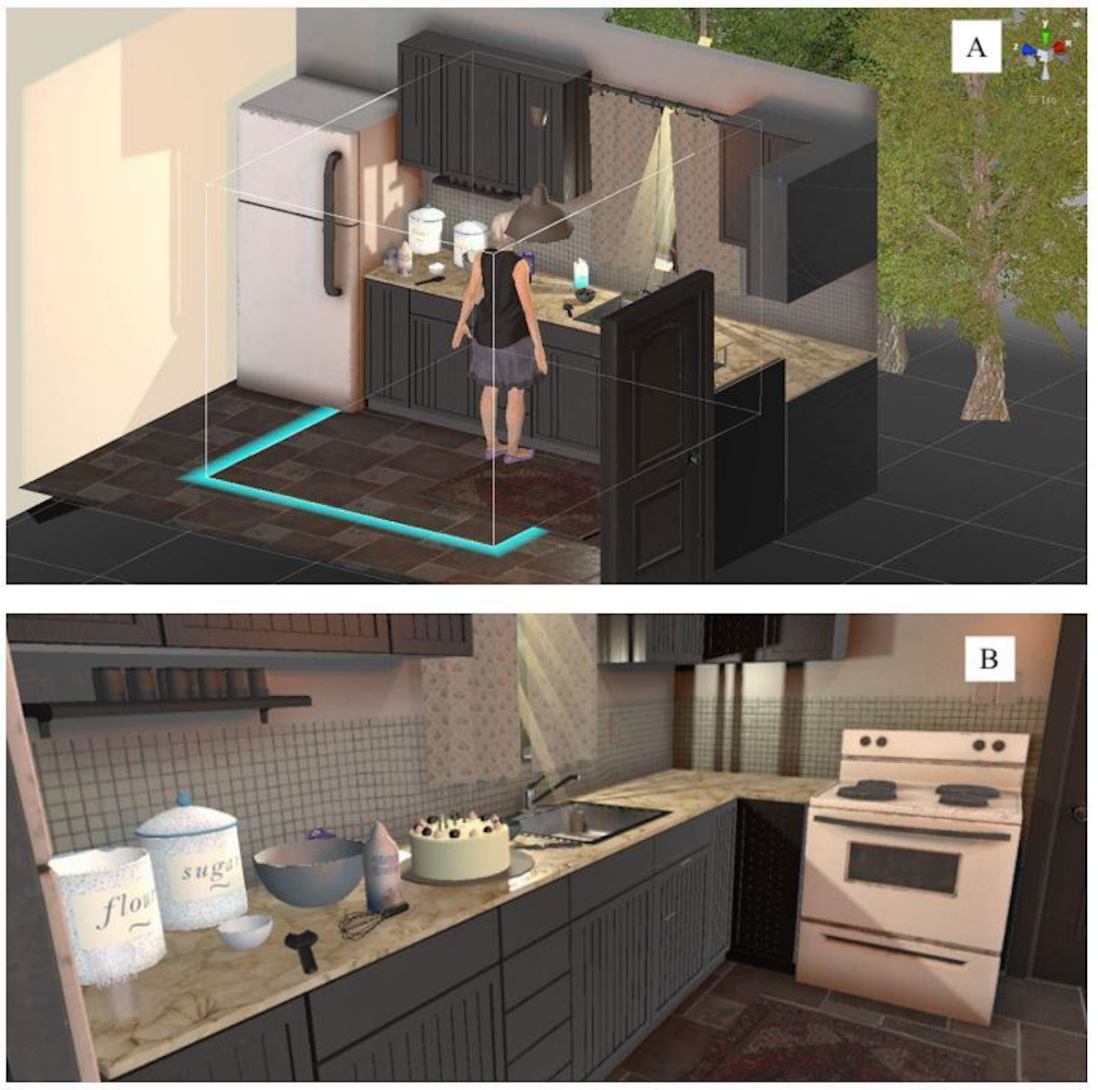
The virtual reality game’s developer view; and the kitchen in *AS IF*.

Now redesigned for a different medium, a major overhaul involved the visual and interactive aspects of the game. However, the narrative of *AS IF* primarily remained the same because participants favored the story and settings. Similar to the initial version, the VR game starts with an introductory tutorial that shows players how to use handheld controllers to interact with *AS IF* and its virtual objects. In contrast to the initial version, the movement tasks of *AS IF* are now *realistic*—direct manipulation of objects by using one’s hands (via controllers). Instead of connecting the dots to get something done, the VR player makes a cake by direct action, such as breaking an egg. In this new version, participants play from a first-person perspective rather than the prior version’s third-person perspective (Figure 1). As the players as grandmother attend to each task of cake making, they simultaneously hear the grandmother’s self-talk: the hopes, frustrations, and fears that stem from the ways chronic pain affects her.

#### Representations of Physical Pain

**Figure 2:**
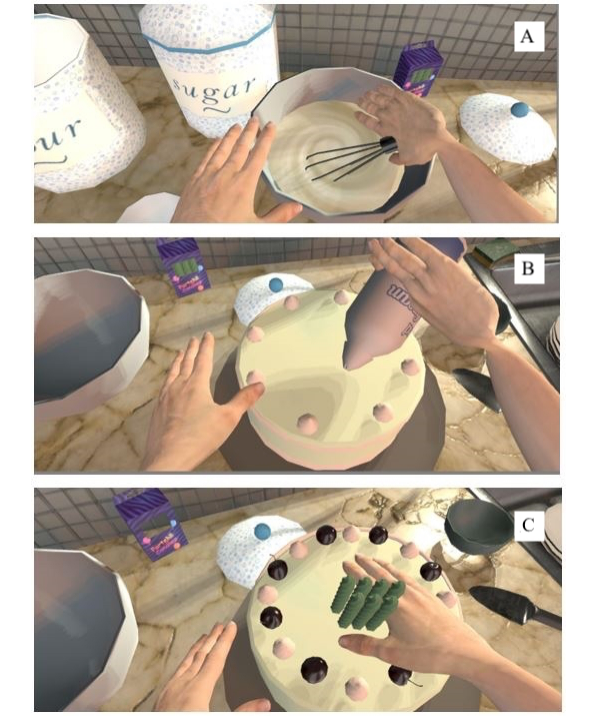
Three selected game tasks in *AS IF*: A. mixing the flour with milk; B. adding cream; and C. adding birthday candles.

**Figure 3:**
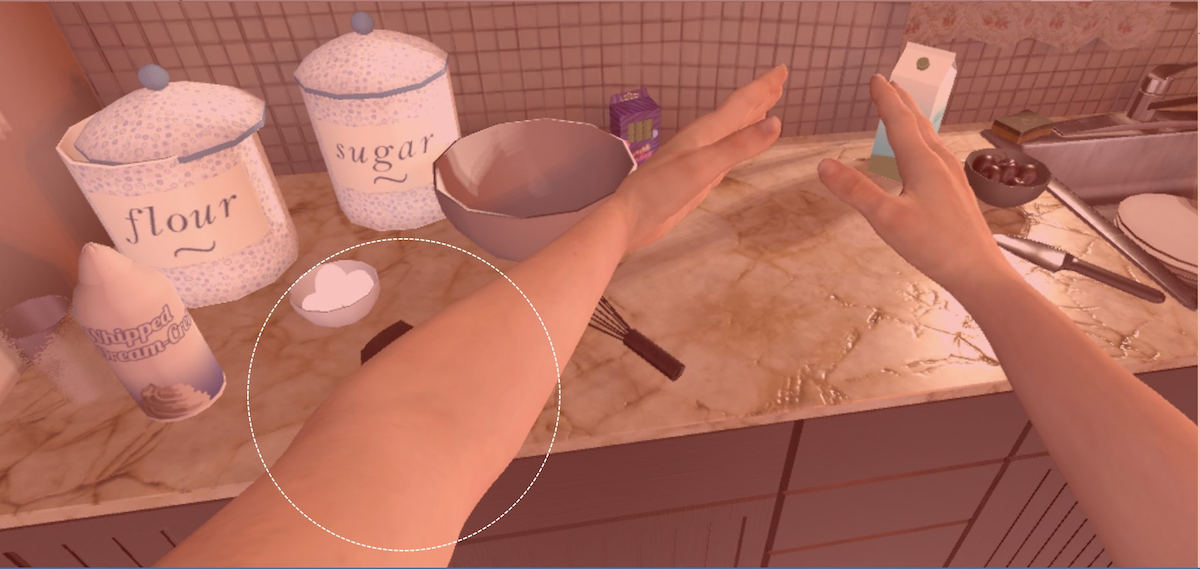
Some of the movements of the virtual avatar’s limbs in *AS IF* become limited when pain increases (as circled in the figure). In addition, red flashes signal the onset of a headache. During the flashes, a transparent red layer appears over the visible areas of the virtual environment.

When the VR player moves each handheld controller, the grandmother avatar (the virtual body inhabited by the player) moves synchronously. A widely adopted *Unity* package named *Final IK* was used to achieve this synchronous movement function [36]. This synchronous movement from a first-person perspective creates an illusion that the player inhabits the avatar—the player thus feels *as if* they are embodied as a grandmother. When the player interacts with the virtual objects, they also experience the avatar’s physical limitations that result from pain. The idea is not to induce pain in the player, but rather to enable the player to get a sense *as if* they are a patient with chronic pain. Here, physical *pain* is made *visible* in 2 ways: by limited movement (range and the ability to hold onto an object; Figure 2) and by visual cues. Another visual effect is that when *pain spikes* in the game, red flashes appear to mimic the onset of a headache (Figure 3). Consequently, these indications of pain are a form of feedback, and players quickly learn that they hinder their ability to accomplish tasks through the avatar.

In general, the overhaul of the VR game *AS IF* is distinguished from prior versions designed to facilitate healthy people’s empathy toward patients in 2 ways. First, the player can now experience, if not physical pain itself, at least a sense of how pain may limit their ability to move and visual representations (red flashes) of an impending headache. To our knowledge, few VR games use visual representations to depict pain, which may limit physical movement and breakthrough pain. Second, the experience of what it may be like to live with chronic pain is implicitly deployed in the virtual environment—players perform direct, realistic tasks as if she/he is the patient with chronic pain; gain a sense of how pain may limit and interfere with ordinary physical movements; see signals that *something is wrong*; and hear self-talk about the fears and anxiety that living with pain can produce.

### Recruitment

Altogether, 18 participants (4 females), aged 19-39 years (mean 24.8, SD 3.8 years), were recruited through convenience sampling method for this study. We placed advertisements in the university campus media and sent emails to the faculty and student groups. The inclusion criterion was anyone older than 19 years, and the exclusion criteria were anyone who had a pain condition or was a pain patient or did not understand English. No participants had a reported history of a chronic pain diagnosis; this was an essential requirement because personal experience of chronic pain could bias expectations of *AS IF*. Nevertheless, 3 participants reported that they had contact with patients with chronic pain.

### Study Intent

The goal of this research study was to determine if a serious VR game such as *AS IF* may influence participants’ empathy for patients with chronic pain (raising awareness and fostering positive attitudes toward patients) and to explore what factors may be important to elicit empathy. Furthermore, based on participants’ feedback on this version of our design, we summarize the fundamental design principles and game components to help guide future works.

### Apparatus

Participants experienced and could interact with objects in the virtual environment via a wired, stereoscopic *HTC VIVE* head-mounted display (HMD) and its handheld controllers. We developed the game using the *Unity3D* game engine, which was responsible for rendering and running the game during the study. These real-time rendered scenes of *AS IF* were sent to the HMD through SteamVR (HTC VIVE) suites; the software was responsible for data between *Unity3D* and the devices via an *API* called *OpenVR*.

### Procedures

A mixed-method design approach was adopted in this pre-test, post-test study. Participants’ empathy levels towards chronic pain patients before and after playing *AS-IF*, and quantitative questionnaires and qualitative interviews were used in parallel to derive the findings [37]. The study lasted for 35 to 40 min in total. On arrival, participants were briefly introduced to the purpose of the study and the entire procedure and were then asked to read and sign the informed consent form. Next, participants were asked to fill out the preintervention questionnaire, which included the *Empathy Scale* (revised from the *Compassion Scale* [38]), the *Willingness to Help Scale*, the VR-adapted *Other in the Self Scale,* and the *Emotional Wheel* evaluation (Multimedia Appendix 1). During the intervention, participants were first shown how to play *AS IF*, and then, they played it for approximately 10-15 min.

When they finished the game, participants were asked to fill out the posttest questionnaires to assess their level of empathy toward patients with chronic pain. The posttest questionnaires were a repeat of the *Empathy Scale*, the *Willingness to Help Scale*, and the *Emotional Wheel* evaluation. The *Other in the Self Scale* was added to assess the *relationship between self and the first-person perspective* in the VR experience (Multimedia Appendix 1). In addition, participants were given a Sense of Embodiment questionnaire to evaluate their perceived level of immersion in the VR game. Finally, through a 15-min semistructured interview, participants discussed their experience, provided feedback, and offered researchers their ideas about the game. The interview topics were primarily about the game’s interactions, depictions of *pain*, and physical impact on participants’ empathetic attitudes in *AS IF*. Meanwhile, the session was audio recorded to ensure that the captured data were accurate.

### Instruments

We used multiple instruments to measure various aspects of empathy. For instance, the *Empathy Scale* (adapted from *Pommier Compassion Scale* [38,39]) measures multiple-dimensioned implicit cognitive changes, whereas the *Willingness to Help Scale* detects explicit cognitive changes. *Wheels of Emotion* reflects emotional changes, and the VR-adapted *Other in the Self* *Scale* assesses the perspective taking aspect of empathy. As chronic pain is a complex process that involves physical, emotional, and social aspects, we adopted instruments that account for cognitive and emotional perspectives regarding pain (refer to Multimedia Appendix 1 for more details on each scale).

To determine which instruments were validated and the most appropriate, we compared existing instruments, such as the *Basic Empathy Scale* [40], *Toronto Empathy Questionnaires* [41], and Baron-Cohen and Wheelwright’s *Empathy Quotient* for people with autism [42]. After thoroughly examining questions from each scale and evaluating how the scales may fit and be adapted for our purposes and population (patients with chronic pain), we selected the *Compassion Scale* [38,39] for its appropriate number and types of questions.

In our previous study of the initial version of *AS IF*, the *Empathy Scale*, the five-point *Numerical Rating Scale* (NRS), and the *Willingness to Help Scale* (10-point NRS) were used to understand if and how that game may have affected participants’ cognitive empathy levels. A total of 2 pretest questionnaires were also used in the previous version: (1) the *Empathy Scale* was used to assess implicit empathy (unconscious emotion) and (2) the *Willingness to Help Scale* was used to assess explicit empathy (conscious emotion).

**Figure 4:**
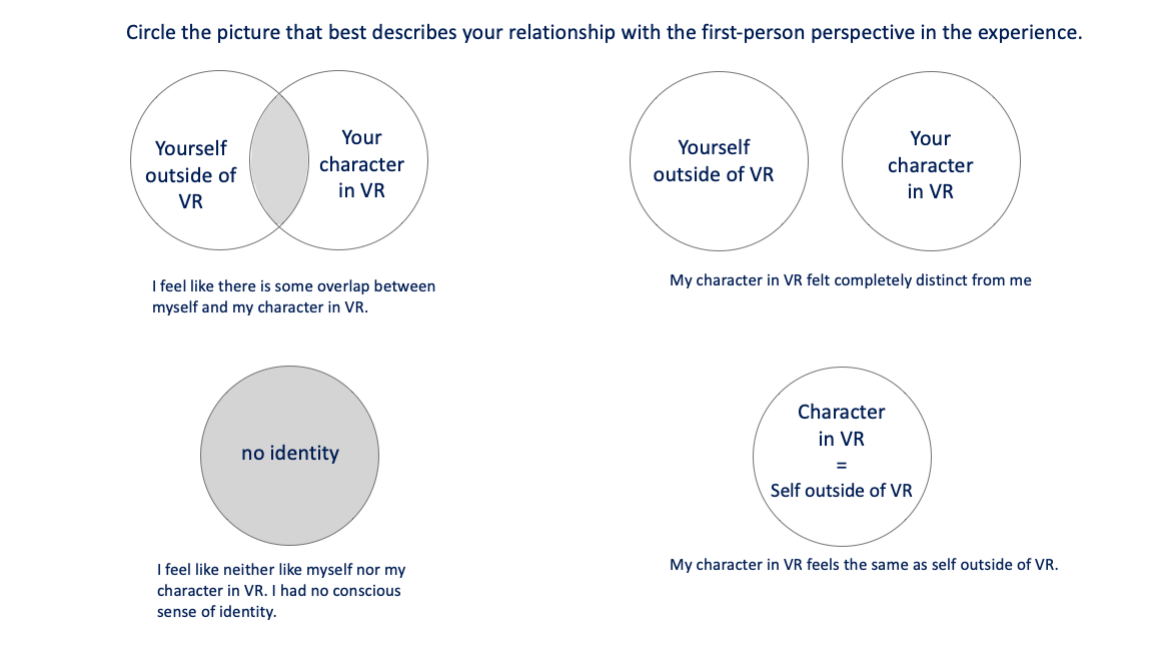
Virtual reality–adapted Other in the Self Scale.

For the new VR version of *AS IF,* the *Wheel of Emotion* [43] and the VR-adapted *Other in the Self Scale* [44] (Figure 4) were adopted to measure any emotional changes associated with empathy and the degree of perspective taking according to the suggestions of Carey et al [45]. The *Wheels of Emotion* provides a comprehensive list of emotions and standardizes the data collected (according to Plutchik’s Psycho-Evolutionary Theory of Emotion), whereas the *Other in the Self Scale* helps players articulate how they related to the grandmother’s avatar. Moreover, to further investigate embodiment in the VR version, we adapted a *sense of ownership (SoO)* and a *sense of agency (SoA)*, rated on an 11-point NRS (from −5 to 5). The adapted questions are listed in Table 1.

**Table 1:** The sense of ownership and sense of agency self-reported questionnaires were sorted by categories.

Embodiment aspects	Questions: while playing *AS IF*
Sense of body ownership	I feel as if the virtual body (eg, arms and hands) were my own (eg, real arms and hands). I feel my real body (eg, arms and hands) were becoming virtual. I feel my real body (eg, arms and hands) were moving sometimes.
Sense of body agency	I feel as if the virtual body (eg, arms and hands) have a will of its own. I feel the virtual body (eg, arms and hands) would move in the same way as my real body (eg, arms and hands).

## Results

### Quantitative Findings

All statistical analyses of the quantitative data were performed using SPSS version 22 (IBM) software [46]. For qualitative data, in-person interviews were first transcribed as electronic textual data. These data were then coded into categories by 2 authors based on preexisting knowledge or hypotheses. After comparing the results, the researchers highlighted significant patterns and summarized and grouped them into themes.

#### Empathy Scale (Adapted Compassion Scale)

**Figure 5:**
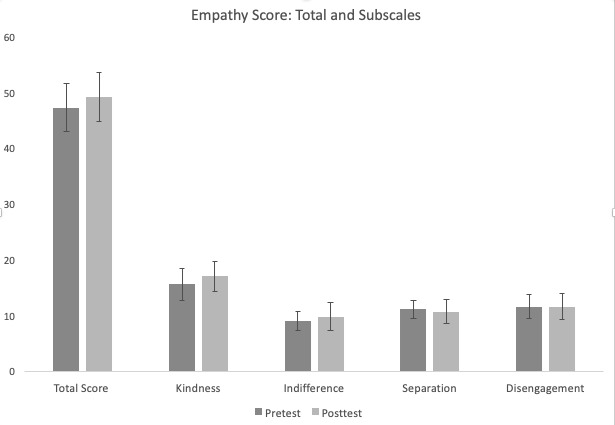
Mean values from the *Empathy Scale* with error bars before and after the study. From left to right: total score, kindness subscale, indifference subscale, separation subscale and disengagement subscale (y-axis: self-reported ratings from the questions in each category).

We used a paired *t* test to analyze the differences between the before and after empathy ratings, total scores and subscale scores (Figure 5), kindness, indifference, separation, and disengagement [38]. For the adapted *Empathy Scale*, the total scores from the pretest (mean 47.33, SD 4.24) and the posttest (mean 59.22, SD 4.33) score did not reach statistical significance (t_17_=−1.41; *P*=.07). However, we found differences in the subscales: the kindness subscale showed a statistically significant increase in the posttest (mean 15.61, SD 2.85) compared with pretest (mean 17.06, SD 2.65; t_17_=−3.97; *P*=.01). However, indifference (t_17_=−1.52; *P*=.14), separation (t_17_=0.75; *P*=.46), and disengagement (t_17_=0; *P*=.99) subscales were not statistically significant before and after the study. The mean and SD values of separation (pretest: mean 11.11, SD 1.64 and posttest: mean 10.72, SD 2.11), disengagement (pretest: mean 11.61, SD 2.17 and posttest: mean 11.61, SD 2.28), and indifference subscales (pretest: mean 9, SD 1.75 and posttest: mean 9.83, SD 2.46) in pretest and posttest are shown in Figure 5. Admittedly, the scores of the overall empathy and its 3 subscales (indifference, disengagement, and separation), which are different combination of questions from the empathy questionnaire, did not change significantly. However, the statistical significance of the kindness subscale revealed that this aspect of empathy could be potentially altered in a VR game such as *AS IF*.

#### Willingness to Help Scale

The *Willingness to Help Scale* has a question that involves a real-world scenario regarding how likely one is to help a patient with chronic pain (refer to Multimedia Appendix 1); it is intended as a means to evaluate the emotional and perspective taking aspects of a participant’s empathy for patients with chronic pain. For the scores of the *Willingness to Help Scale* before and after the game intervention and from a *t* test analysis, a significant increase in the posttest score was observed (mean 8.33, SD 2.03) compared with the pretest score (mean 7.17, SD 2.28; t_17_=−4.51; *P*<.001). The effect size for this analysis was found to be large, according to Cohen convention (*d*=−1.063). This statistically significant increase indicates that the game was able to increase participants’ explicit willingness to help people with chronic pain.

#### The Wheel of Emotions

In their protocol paper for measuring empathy in VR, Carey et al [45] recommended using the *Wheel of Emotions* *Scale* to measure the emotional aspects of empathy. Specifically, this instrument is intended for understanding emotional empathy or *the spontaneous feeling of oneness with another’s emotions* [45]. Therefore, we reported the basic analysis of each participant’s emotional changes before and after the study. Overall, 12 of the 18 participants changed from positive emotions (eg, joy, love, and optimism) to negative emotions (eg, sad, helpless, and scared). This may have been influenced by the *AS IF* experience. Six participants reported no changes, regardless of what their initial emotions were. However, half of the 6 participants first described their emotions as negative ones (eg, sad, helpless, and scared). Therefore, most participants’ emotions appeared to have changed from a positive to negative direction. Given that the virtual character’s self-talk can be characterized by a sense of frustration and fear, these results suggest that *AS IF* fostered emotional empathy.

#### VR-Adapted Other in the Self Scale

A fourth set of tools was needed to understand cognitive empathy, characterized by understanding another’s perspective while also maintaining a distinct sense of self. Regarding the relationship between the virtual avatar and the participant’s self, 13 of the 18 participants reported feeling an overlap between their sense of self and the virtual avatar. Three participants felt completely distinct from the avatar, one participant reported not feeling any identity of himself (either inside or outside the game), and one participant reported feeling completely the same. Therefore, 14 of the 19 participants (74%) could relate the virtual avatar to themselves while playing *AS IF.* In general, the results from the *VR-adapted* *Other in the Self Scale* suggest that most participants felt the virtual body overlapped with their real identity—the perspective taking aspect of empathy. Therefore, most participants were able to take the perspective of the grandmother who suffers from chronic pain in *AS IF*.

#### Sense of Embodiment—the SoO and the SoA

As one of the goals was to investigate whether embodiment in VR affected or correlated with changes in empathy, we collected posttest data regarding the participants’ SoO (of the avatar) and SoA in VR. On average, participants’ scores were higher than zero for both SoO (mean 1.28, SD 2.78) and SoA (mean 1.5, SD 2.65). In the SoO and SoA questionnaires (Table 1) because the rating scale ranged from −5 to 5 (−5 means strongly disagree, and 5 means strongly agree with the statements), the mean values here show that the participants experienced a medium to slightly strong level of body ownership and agency over the virtual avatar.

#### Correlation Analysis

Pearson correlation tests were also run to test the relationship between SoO and SoA, between the sense of embodiment (comprising SoO and SoA) and the Willingness to Help Scale, and between the sense of embodiment and the *Empathy* Scale (comprising 4 subscales). The results show that SoO is significantly correlated to SoA (r18=0.832; *P*<.001), indicating that the participants’ SoO strongly correlates with their SoA in AS IF. Although the correlation between SoO and *Empathy Scale*scores (*P*=.10) and the SoA and Empathy Scale (*P*=.11) did not reach statistical significance, the *P* values fell just short of statistical significance. Finally, the results from the Willingness to Help Scale had statistically significant positive correlations with the kindness subscale (r18=0.632; *P*=.005) and statistically negative correlations with the indifference subscale (r18=−0.531; *P*=.02).

### Qualitative Findings

Here, we discuss what the participants thought of *AS IF* and evaluate the strengths and weaknesses of the VR game. We also discuss which of the game’s main features may be useful for future research.

#### Participants’ Views About the AS IF VR Game

Although *AS IF* does not simulate the physical feeling of persistent pain, the game achieved its primary goals: to motivate the participants to reflect on experiences of patients with chronic pain and to raise empathy.

One participant said:

I think this can help me to understand more about patients. It definitely made me start thinking about how hard other day-to-day tasks would be for people with chronic pain.

P19

Overall, participants considered the game interaction to be easy to follow and very intuitive. For instance, P05 said:

The interaction was pretty good and very illustrative.

P08 and P09 said:

The interaction is pretty straightforward.

The 2 approaches of representing pain in VR had pros and cons in providing an immersive experience of a patient with chronic pain*.* Nonetheless, the VR game shows a high potential for fostering cognitive and emotional empathetic attitudes toward people with chronic pain.

#### Representing Pain, Approach 1: Restricting Movement to Represent Pain

In general, none of the participants had trouble understanding or completing the VR game’s tasks. From the interviews, most participants were aware that the physical limitations imposed in VR represented pain. However, a few participants initially reported that these restrictions felt more like a bug in the program. Overall, approximately one-third of the participants considered the randomly frozen hands/arms to be annoying and more like technical issues. For instance, P08 and P10 told us that:

At first, I thought the arms had some delay compared to my real arms, and I thought it was technical difficulties. Then I realized it was the game setting.

Nonetheless, to a certain degree, this mechanism did achieve the goal because participants’ emotional status changed, leading to an increased empathetic attitude toward the grandma patient. Overall, 14 participants reported that their affective changes emerged because of the game and described it using negative emotional words, including *depressed*, *impatient*, *upset*, *frustrated*, *pensive*, *sorry*, and *pity*. For instance, P01, P03, P04, and P11 said:

I felt lonely in the game and frustrated while playing the game, but in the end, I am happy to finish the game and achieve the patient’s goal [cake making].

P01

I felt like my movements were slowed down. Plus, I made a mess in the kitchen by dropping things. Emotionally, it was a little discouraging and lowered my confidence with being able to bake all by myself.

P04

It was pensive. I was thinking like people with chronic pain, how it’s gonna be for them.

P11

The participants reported that their empathy was elicited when they felt that they were incapable of handling easy daily activities as a virtual patient. P10 said:

Suffering pain and I should take a rest and slow down my movement later. I feel that everyone else could make a cake faster than me.

Although 3 of the participants said they did not have emotional feelings about the grandmother, all agreed that this VR game brought about an awareness. For the first time, participants said they started to think about what life would be like for patients with chronic pain. For instance, P16 said:

Although I can’t feel any pain, I can feel the difficulty of [the] tasks.

Interestingly, some participants offered suggestions for improving the game, such as adding more and different forms of feedback regarding movement restrictions, such as a pain meter or digital pain diary. Some participants also suggested that if the granddaughter was visible, she could provide contrast with the physical problems the grandma experiences because of her chronic pain. For instance, P09 said:

The limited movement also helps as I cannot move quickly, which is appropriate for a grandma at her age. But, it would be better if there is any contrast, for instance, having a very active child, or people who can move fast.

#### Representing Pain, Approach 2: The Red Flashes Signaling the Pain of a Headache

Generally, participants’ responses to the red flash effect matched our expectations. Specifically, the red flashes elicited some sense of *pain*, if only vaguely, through a visual effect. In total, participants reported 3 types of sensations when they saw this visual stimulus. The first type reported by the majority of participants was that the reddened world made them feel *pain* and *headaches*.

For example:

I did notice the red filter effect, and I can understand that the game was trying to express what the pain patient may have experienced.

P03

It was very annoying, and I cannot think or move when the red shadow happens.

P05

I feel very dizzy when the red flashes were coming out, and I cannot think anymore. My brain was entirely blank, and this can definitely represent pain to me.

P08

The second type of sensations participants experienced in response to the red flashes were not physical pain per se, but an idea of what the reddened environment was meant to be, and they felt bothered by it. For example, 2 participants mentioned that although they did not feel physiological pain:

It can work as an indication to slow down my movement...but I do not feel any physical pain myself.

P09

The third type of response to the red flashes was an inability to make an association between the visual indication of an impending headache (or pain spike) with the patient’s pain experience. Two participants reported that they did not understand this idea and did not think it represented pain effectively.

#### The Narrative Strengthened the Sense of Immersion

From participants’ feedback, the realistic visual depiction of the kitchen and cake making tasks strengthened the experience of *being* a patient with chronic pain:

Everything in this game was so realistic and well-done. I was beginning to embody myself to the character and feel I was there. There were moments that I forgot that was me.

P07

In addition to the visual simulation, the audio of the grandmother’s inner voice or self-talk also strengthened the sense of immersion and empathy. For instance:

It could put me into this situation by narrating that for me.

P18

Yes, the narrator was expressive, and the voice felt very exhausted and tired. I think the audio was the most influential part and it directed the story.

P13

The most significant change in the new VR version of *AS IF* was that tasks are now accomplished directly, rather than indirectly by solving puzzles. In the new VR version, participants complete the tasks of baking a cake by directly interacting with virtual objects, such as stirring together ingredients in a bowl, just as they would in the real world; this also increased the sense of immersion. Moreover, the ordinariness or daily life aspects of the tasks also appear to have succeeded in raising awareness of what life with chronic pain might be like.

As P19 said:

Normally, speaking of chronic pain patients, I usually think of the hospital or [them] laying on [a] bed. Baking a cake bring[s] me more awareness about how daily life could be so hard for them too. I won’t feel having empathy for them if not doing these tasks. Right now, I feel more related to the grandma in the game.

Therefore, providing a connection to the virtual avatar—by performing realistic tasks and multimodal sensory input in VR—better situated the participants *as if* they were *in the patients’ shoes*. P11 mentioned that he was thinking of his mother’s chronic pain while inhabiting the grandma avatar. He felt frustrated, and his experience in *AS IF* reminded him how hard his mother’s life was.

## Discussion

### Principal Findings

We explored a significant redesign and study results of a VR game, the *AS IF*. It is aimed at motivating *people who do not live with chronic pain* (nonpatients) to better understand the lived experience of chronic pain by increasing empathy. In general, the findings demonstrate that participants had greater degrees of empathy toward patients after playing the VR game. Furthermore, from the semistructured interviews, we were able to gather essential feedback about the strengths and limitations of the current VR design, such as the effectiveness of pain representations. Finally, we extracted critical design issues, implications, and protocol suggestions and offered them to potentially benefit similar research in the future.

Overall, after playing the VR game *AS IF,* participants scored significantly higher on the *Willingness to Help Scale* and the kindness subscale—an adaptation of the *Empathy* questionnaire. These two scores revealed that not only could one VR experience of *AS IF* raise people’s awareness of chronic pain but it could also increase their *implicit* and *explicit* empathy. Data from the *kindness* subscale showed implicit cognitive changes, whereas data from the *Willingness to Help* *Scale* revealed changes in explicit empathic attitudes toward patients with chronic pain. The other three subscales showed nonsignificant differences. Furthermore, the qualitative interview data show that most participants reported that playing this game helped them to understand what a chronic pain patient’s life would be like, and that they had never thought about that before. We assume these findings may result from 2 potential reasons: (1) indifference, disengagement, and separation are difficult to affect or change during a single, short period, as in this study, and (2) the design of the VR game *AS IF* focused more on the perspective taking and emotional aspects of empathy, but it did not have specific game features that were meant to increase the four subscales.

The *VR-adapted* *Other in the Self Scale* suggested that most participants felt that the virtual body overlapped with their real identity. The findings from the *VR-adapted Other in the Self Scale* also overlapped with the interview results. Some participants said they felt embodied in the grandma avatar who has chronic pain through narrative storytelling, the immersive environment, and the game tasks. Thus, in *AS IF* game*,* participants were able to understand the perspective of the grandma who has chronic pain.

The sense of embodiment scores showed that, on average, participants could sense owning and controlling the virtual avatar. However, the *Pearson* correlation test revealed no statistical significance between the sense of embodiment in VR (comprising SoO and SoA) and the *Empathy Scale* (posttest) or the sense of embodiment in VR and the *Willingness to Help* scale. We conjecture that 2 reasons might account for this nonsignificant outcome. First, there could be multiple factors that affected empathy levels besides embodiment, such as the narrative and the game’s specific tasks (and the *fun/frustration* behind that). Hence, a single factor might not be strong enough to alter overall feelings of empathy. As mentioned in the interviews, participants suggested that tactile feedback might be a better way to indicate pain or the association of visual effects with pain. The second possible explanation could be that the game did not provide a strong enough sense of embodiment to reach statistical significance. In interviews, participants said they wanted the virtual avatar to more closely match their own gender and ethnicity and perhaps even body height and shape. A few participants—a male and a participant whose skin color differs from the avatar’s—reported they felt disembodied with the virtual avatar because of its divergent characteristics.

Admittedly, the overall empathy scores and the 3 subscales (indifference, disengagement, and separation) did not change significantly. A crucial issue is how long it takes to affect and change empathy and what factors are important in facilitating such change. For example, implicit empathy may be difficult to change in a short time, in part because of its mental cost [47]. However, in this study, the *VR-adapted Other in the Self* Scale findings suggest that participants identified with the VR avatar, insofar as the avatar was felt to overlap with their real self. Therefore, a perspective taking ability may be critical to being able to influence one’s empathetic attitudes toward patients with chronic pain and *painful* experiences. From the qualitative interviews, 2 approaches to representing pain in the virtual body may also facilitate empathy. Although a few participants found the movement restrictions confusing, most reported it made them realize how pain would impact one’s range of motion and emotion. Most liked the idea of using the *red flashes* to represent pain spikes and reported that the visual effect felt like a headache or pain (a synesthesia effect of transferring a visual sense to an emotional sense).

Besides facilitating nonpatients’ (game players who are health care givers or family members of patients with chronic pain) empathy toward patients with chronic pain, we believe that a VR game such as *AS IF* has the potential to benefit patients and other researchers. Chronic pain experiences are notoriously difficult to describe and are often out of the experiential scope for most people. Hence, VR is one method that may provide clinicians and family members or friends who play a caregiving role with a deeper understanding of a patient’s chronic pain condition. VR appears to have the potential to change the player’s mind significantly [48] and to stimulate perspective taking and/or behavioral changes that are associated with empathy toward others like patients [49,50]. For instance, in previous research, Platt et al [51] showed how empathic communication, such as clinicians’ awareness of the patients’ affective states and showing appreciation of the patient’s feelings, may reduce patients’ feelings of isolation [43]. In addition, the findings, experiences, and design suggestions from this paper may directly benefit other researchers in the future developing empathic games for patients with chronic pain specifically or for patients who must manage similarly invisible chronic conditions. Given our aging population, this may be particularly useful as an approach to medicine shifting from treating acute conditions to managing chronic conditions and promoting wellness.

Some of the participants questioned, “Was my pain sensation the chronic patients’ pain too?” Although it is impossible to make this determination in this study, we assume that the sensations were not the same. For one thing, chronic pain is unique and difficult to describe, let alone recreate specific perception. For another, the interview findings suggested that current gameplay increased the participants’ awareness of chronic pain patients’ situation through the narrative, visual, and audio feedback in *AS IF*.

### Limitations

This study also had a few limitations that may affect the empathy outcomes and bring a risk of bias regarding the conclusions. First, the sample size for this preliminary study was small, and a larger number of clinicians will be tested once the prototype is revised and prepared for clinical deployment. Next, to avoid overwhelming participants with assessment instruments, the level of immersion in VR was not measured, so we do not know if there are potential relationships between immersion and changes in empathy. Moreover, we only conducted a preliminary, in-laboratory study using questionnaires and interviews to evaluate changes in empathy, and no real-life assessment has been implemented. However, evaluating the pragmatic aspects of the VR game is definitely something we are planning. This involves running a practical test immediately after the subsequent study, asking participants to donate a portion of their study compensation to a hospital’s foundation or a nonprofit organization for patients with chronic pain. Finally, we did not have a control group and did not conduct a follow-up study to see if any long-term empathy behavioral changes persisted. Investigating these factors to determine whether they affect changes in empathy are important next steps.

### Comparison With Prior Work

For a long time, researchers have been looking for evidence about how the sense of embodiment in VR may impact one’s cognitive perception [48]. Various potential impact factors of virtual embodiment on empathy (perspective taking) have been investigated, notably, attitudes toward racial bias [52], gender bias [53], and age [54,55]. We explored the relationship between SoO, SoA, and empathy through correlation tests. However, although participants reported a medium-level SoO and SoA over the virtual body, no significant relationship was discovered.

In summary, to put nonpatients *in the shoes of* patients with chronic pain, players *inhabit* a virtual body of a patient with chronic pain who attends to everyday tasks in the VR game *AS IF*. It simulates several experiences common to chronic pain: physical limitations of movement and a patient’s verbally articulated self-talk. The visual-motor synchronicity of a player’s full-body movements mirrored by the avatar appears to elicit identification with the avatar. The results from the mixed methods study revealed that the game was effective in improving implicit and explicit empathy. Furthermore, the findings showed that the game raised the emotional and perspective taking aspects of players’ empathy. However, no associations were found between the sense of embodiment (SoO and SoA) and the empathy scales in this game. On the basis of the analysis of participants’ feedback, we developed and proposed design suggestions for empathic games designed to facilitate understanding of pain patients. In future work, we plan to iterate the design features and study protocols according to participants’ feedback. We also plan to conduct a randomized controlled study with a larger sample size that is more diverse in terms of gender and age. As for the game, we plan to implement tactile feedback in the controllers (or body sensors) that matches the game tasks, and we would also prepare virtual avatars of different genders and ethnicities.

## Appendix

Multimedia Appendix 1 Study questionnaires, interview guide, and correlational tables.

## Abbreviations

HMD: head-mounted display
NRS: Numerical Rating Scale
SoA: sense of agency
SoO: sense of ownership
VR: virtual reality
