# Supplementary material for: Designing a Virtual Reality Game for Promoting Empathy Toward Patients With Chronic Pain: Feasibility and Usability Study
Source: JMIR Serious Games. 2020 Aug 7;8(3):e17354. doi: 10.2196/17354 (PMC7442937; doi:10.2196/17354)

**Supplementary Material**

**1. Pretest Questionnaire**

[Note: the following 16-item questionnaire is based on the “Compassion Scale” (Pommier, 2011), modified for Chronic Pain]

HOW I TYPICALLY ACT TOWARDS **P**EOPLE **W**ITH **C**HRONIC **P**AIN (PWCP)*

* Chronic Pain is defined as any pain that lasts longer than six months. With chronic pain, signals of pain remain active in the nervous system for months or even years. This can take both a physical and emotional toll on a person.

Please read each statement carefully before answering. To the left of each item, indicate how often you behave in the stated manner, using the following scale:

1. If PWCP cry in front of me, I often don’t feel anything at all.

Almost                                                                                                     Almost

Never                                                                                                       Always

**1                             2                             3                             4                             5**

2. Sometimes if PWCP talk about their problems, I feel like I don’t care.

Almost                                                                                                     Almost

Never                                                                                                       Always

**1                             2                             3                             4                             5**

3. I don’t feel emotionally connected to people in chronic pain.

Almost                                                                                                     Almost

Never                                                                                                       Always

**1                             2                             3                             4                             5**

4. I feel detached from PWCP if they tell me their tales of woe.

Almost                                                                                                     Almost

Never                                                                                                       Always

**1                             2                             3                             4                             5**

5. If I see someone with chronic pain going through a difficult time, I try to be caring toward that person.

Almost                                                                                                     Almost

Never                                                                                                       Always

**1                             2                             3                             4                             5**

6. I will tune out if PWCP tell me about their troubles.

Almost                                                                                                     Almost

Never                                                                                                       Always

**1                             2                             3                             4                             5**

7. I will like to be there for PWCP in times of difficulty.

Almost                                                                                                     Almost

Never                                                                                                       Always

**1                             2                             3                             4                             5**

8. If I see PWCP feeling down, I feel like I can’t relate to them.

Almost                                                                                                     Almost

Never                                                                                                       Always

**1                             2                             3                             4                             5**

9. Sometimes I am cold to PWCP if they are down and out.

Almost                                                                                                     Almost

Never                                                                                                       Always

**1                             2                             3                             4                             5**

10. I don’t concern myself with PWCP’s problems.

Almost                                                                                                     Almost

Never                                                                                                       Always

**1                             2                             3                             4                             5**

11. My heart goes out to PWCP who are unhappy.

Almost                                                                                                     Almost

Never                                                                                                       Always

**1                             2                             3                             4                             5**

12. If I saw PWCP are feeling troubled, I usually will let someone else attend to them.

Almost                                                                                                     Almost

Never                                                                                                       Always

**1                             2                             3                             4                             5**

13. I don’t think much about the concerns of PWCP.

Almost                                                                                                     Almost

Never                                                                                                       Always

**1                             2                             3                             4                             5**

14. I can’t really connect with other PWCP when they’re suffering.

Almost                                                                                                     Almost

Never                                                                                                       Always

**1                             2                             3                             4                             5**

15. I try to avoid PWCP who are experiencing a lot of pain.

Almost                                                                                                     Almost

Never                                                                                                       Always

**1                             2                             3                             4                             5**

16. If PWCP feel sadness, I try to comfort them.

Almost                                                                                                     Almost

Never                                                                                                       Always

**1                             2                             3                             4                             5**

*The following question ask you to imagine a scenario and what you would do accordingly.*

You’re preparing for an important interview for tomorrow. However, a friend who have chronic pain ask you if you can help with a ride to airport (a 2 hour drive each way). Please indicate how willing are you to help the person?

             1   2 3   4 5 6   7 8 9 10

Not at all                                            Very willing to help

Please circle the emotions that you feel before playing this game:

**
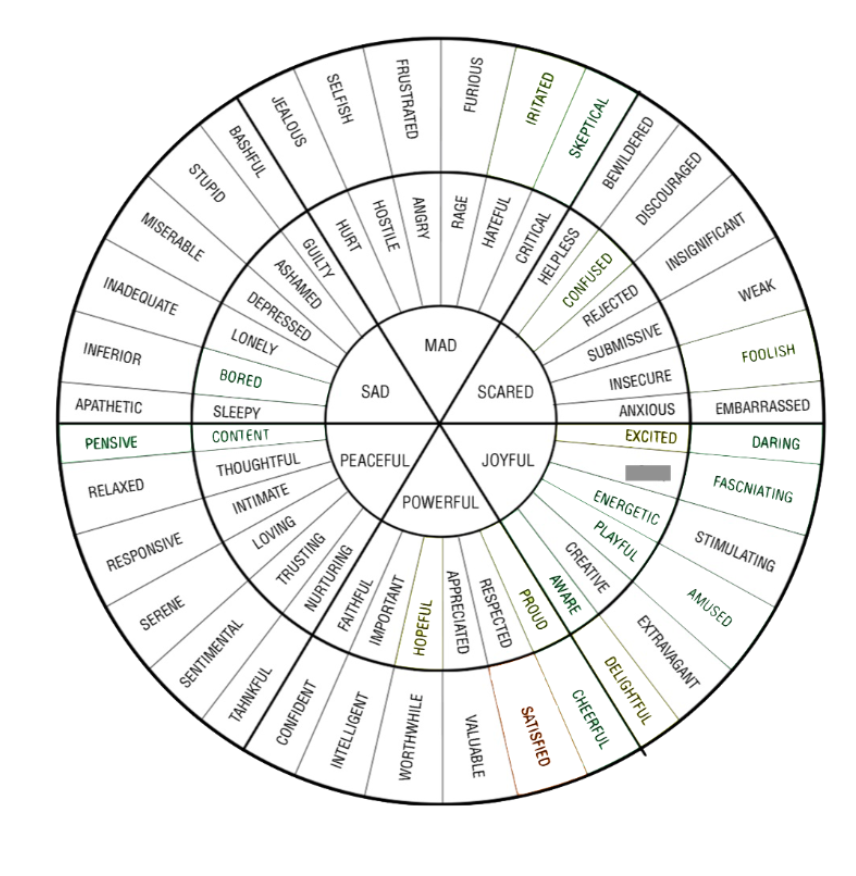
**

*The following questions will ask you some personal information regarding chronic pain and demographic information. Your answer is completely anonymous. And we will keep these information in secure condition.*

Do you know of any families or friends who had ever been diagnosed with chronic pain?

1. Yes         b. No

Have you ever been diagnosed with chronic pain before?

1. Yes          b. No

Could you tell us your age?

_______

Could you tell us your gender?

1. Female                 b. Male c. Other        d. Prefer not to disclose

**2. Post-test Questionnaire**

HOW I TYPICALLY ACT TOWARDS **P**EOPLE **W**ITH **C**HRONIC **P**AIN (PWCP)

Please read each statement carefully before answering. To the left of each item, indicate how often you behave in the stated manner, using the following scale:

1. If PWCP cry in front of me, I often don’t feel anything at all.

Almost                                                                                                     Almost

Never                                                                                                       Always

**1                             2                             3                             4                             5**

2. Sometimes if PWCP talk about their problems, I feel like I don’t care.

Almost                                                                                                     Almost

Never                                                                                                       Always

**1                             2                             3                             4                             5**

3. I don’t feel emotionally connected to people in chronic pain.

Almost                                                                                                     Almost

Never                                                                                                       Always

**1                             2                             3                             4                             5**

4. I feel detached from PWCP if they tell me their tales of woe.

Almost                                                                                                     Almost

Never                                                                                                       Always

**1                             2                             3                             4                             5**

5. If I see someone with chronic pain going through a difficult time, I try to be caring toward that person.

Almost                                                                                                     Almost

Never                                                                                                       Always

**1                             2                             3                             4                             5**

6. I will tune out if PWCP tell me about their troubles.

Almost                                                                                                     Almost

Never                                                                                                       Always

**1                             2                             3                             4                             5**

7. I will like to be there for PWCP in times of difficulty.

Almost                                                                                                     Almost

Never                                                                                                       Always

**1                             2                             3                             4                             5**

8. If I see PWCP feeling down, I feel like I can’t relate to them.

Almost                                                                                                     Almost

Never                                                                                                       Always

**1                             2                             3                             4                             5**

9. Sometimes I am cold to PWCP if they are down and out.

Almost                                                                                                     Almost

Never                                                                                                       Always

**1                             2                             3                             4                             5**

10. I don’t concern myself with PWCP’s problems.

Almost                                                                                                     Almost

Never                                                                                                       Always

**1                             2                             3                             4                             5**

11. My heart goes out to PWCP who are unhappy.

Almost                                                                                                     Almost

Never                                                                                                       Always

**1                             2                             3                             4                             5**

12. If I saw PWCP are feeling troubled, I usually will let someone else attend to them.

Almost                                                                                                     Almost

Never                                                                                                       Always

**1                             2                             3                             4                             5**

13. I don’t think much about the concerns of PWCP.

Almost                                                                                                     Almost

Never                                                                                                       Always

**1                             2                             3                             4                             5**

14. I can’t really connect with other PWCP when they’re suffering.

Almost                                                                                                     Almost

Never                                                                                                       Always

**1                             2                             3                             4                             5**

15. I try to avoid PWCP who are experiencing a lot of pain.

Almost                                                                                                     Almost

Never                                                                                                       Always

**1                             2                             3                             4                             5**

16. If PWCP feel sadness, I try to comfort them.

Almost                                                                                                     Almost

Never                                                                                                       Always

**1                             2                             3                             4                             5**

*The following question ask you to imagine a scenario and what you would do accordingly.*

You’re preparing for an important interview for tomorrow. However, a friend who have chronic pain ask you if you can help with a ride to airport (a 2 hour drive each way). Please indicate how willing are you to help the person?

             1   2 3   4 5 6   7 8 9 10

Not at all                                            Very willing to help

Please circle the emotions that you feel after playing this game:

**
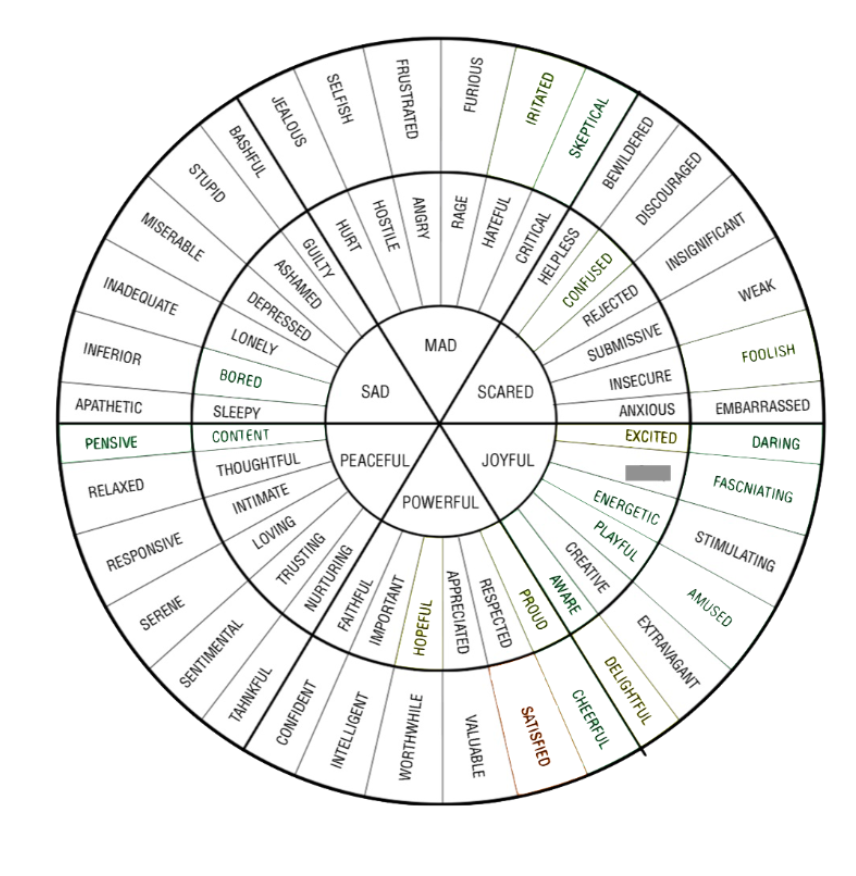
**

**
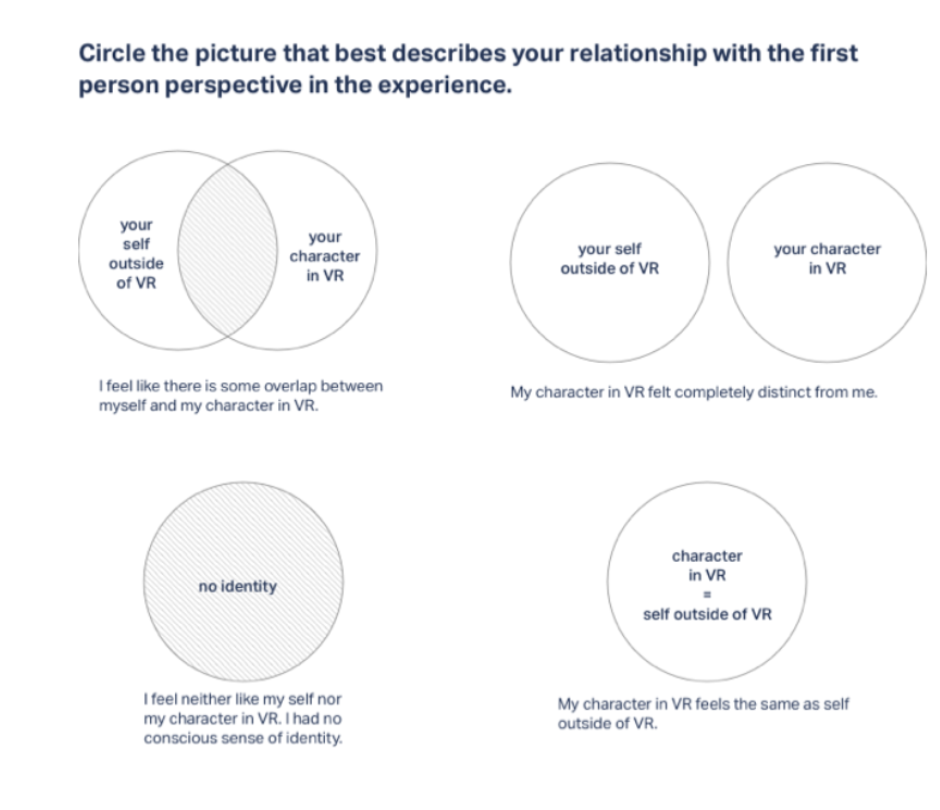
**

**3. Post-test semi-structured Interview Guide**

Do you have any difficulties while playing the game? If so, what they are?

What do you think about the game interaction, such as controlling your avatar or interacting with the game objects, connecting the dots, etc.?

Can you describe how the virtual physical limitations on your avatar made you feel?

While playing the game, what were your physical sensations, if any?

While playing the game, what were your emotional experiences, if any?

How do you feel about the “fisheye” lens effect and glowing limbs as a visual representation for chronic pain?

In your opinion, does the game reflect the real patient’s sufferings, i.e.: put you into the shoes of chronic pain patients? Why or why not?

How would you design or redesign this game if you were the game designer?

*That is the end of study. Thank you very much for your participation!*

**4. Correlation Tables**

4.1 Sense of ownership and related factors correlation table (* means significant level < .05, and ** means significant level < .01)

| Factor | Pearson Correlation | Significant |
| --- | --- | --- |
| Agency | .832** | .000 |
| Empathy Scale Total Score (adapted Compassion Scale) in Post Test | -.397 | .103 |
| Kindness Subscale in Post Test | .115 | .649 |
| Indifference Subscale in Post Test | -.125 | .621 |
| Separation Subscale in Post Test | -.445 | .064 |
| Disengagement Subscale in Post Test | -.342 | .165 |

4.2 Sense of agency and related factors correlation table (* means significant level < .05, and ** means significant level < .01)

| Factor | Pearson Correlation | Significant |
| --- | --- | --- |
| Ownership | .832** | .000 |
| Empathy Scale Total Score (adapted Compassion Scale) in Post Test | -.389 | .110 |
| Kindness Subscale in Post Test | -.172 | .495 |
| Indifference Subscale in Post Test | .054 | .831 |
| Separation Subscale in Post Test | -.216 | .390 |
| Disengagement Subscale in Post Test | -.399 | .101 |

4.3 Willingness to Help scale and related factors correlation table (* means significant level < .05, and ** means significant level < .01)

| Factor | Pearson Correlation | Significant (2-tailed) |
| --- | --- | --- |
| Ownership | .296 | .234 |
| Agency | .153 | .544 |
| Empathy Scale Total Score (adapted Compassion Scale) in Post Test | -.042 | .867 |
| Kindness Subscale in Post Test | .632^**^ | .005 |
| Indifference Subscale in Post Test | -.531^*^ | .023 |
| Separation Subscale in Post Test | -.156 | .537 |
| Disengagement Subscale in Post Test | -.098 | .700 |

5. Supplementary Figure 1. Distribution of the participants’ posttest ratings in the *Virtual Reality–adapted Other in the Self Scale* in (y-axis: the number of participants; see Figure 4 for the questionnaire). VR: virtual reality.


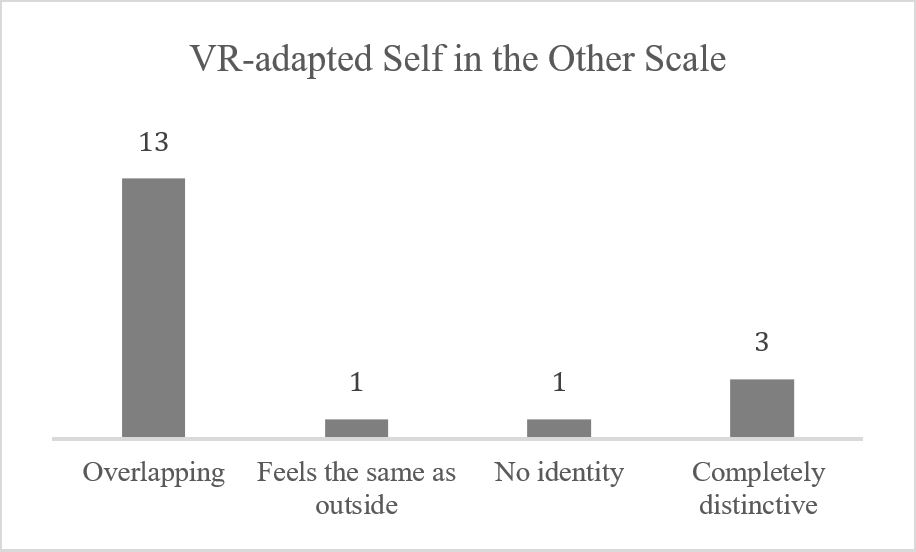

Supplement: Multimedia Appendix 1 [file games_v8i3e17354_app1.docx]
